# Criminal Responsibility and Neuroscience: No Revolution Yet

**DOI:** 10.3389/fpsyg.2019.01406

**Published:** 2019-06-27

**Authors:** Ariane Bigenwald, Valerian Chambon

**Affiliations:** ^1^Département de Philosophie, Université Paris I Panthéon Sorbonne, Paris, France; ^2^Institut Jean Nicod (ENS – EHESS – CNRS), Département d’Etudes Cognitives, Ecole Normale Supérieure, PSL University, Paris, France

**Keywords:** criminal responsibility, liability, free will, sense of agency, neuroscience, neurolaw, cognitive bias, moral agent

## Abstract

Since the 1990’s, neurolaw is on the rise. At the heart of heated debates lies the recurrent theme of a neuro-revolution of criminal responsibility. However, caution should be observed: the alleged foundations of criminal responsibility (amongst which free will) are often inaccurate and the relative imperviousness of its real foundations to scientific facts often underestimated. Neuroscientific findings may impact on social institutions, but only insofar as they also engage in a political justification of the changes being called for, convince populations, and take into consideration the ensuing consequences. Moreover, the many limits of neuroscientific tools call for increased vigilance when, if ever, using neuroscientific evidence in a courtroom. In this article, we aim at setting the basis for future sound debates on the contribution of neuroscience to criminal law, and in particular to the assessment of criminal responsibility. As such, we provide analytical tools to grasp the political and normative nature of criminal responsibility and review the current or projected use of neuroscience in the law, all the while bearing in mind the highly publicized question: can neuroscience revolutionize criminal responsibility? Answering this question implicitly requires answering a second question: *should* neuroscience revolutionize the institution of criminal responsibility? Answering both, in turn, requires drawing the line between science and normativity, revolution and dialogue, fantasies and legitimate hopes.

## Introduction

“A truly scientific, mechanistic view of the nervous system make[s] nonsense of the very idea of responsibility” states Dawkins, a biologist for whom neuroscience would overthrow the retributivist foundations of criminal law (Dawkins, 2006). Others abound in his direction: supporting that “free will is an illusion,” Greene and Cohen (2004) wish to replace retribution with deterrence, prevention and medical treatment. In a similar vein, Sapolsky upholds “a world of criminal justice in which there is no blame, only prior causes” (Sapolsky, 2004).

Neuroscience and indeed all disciplines studying brain structure and function have had a growing influence on political discourse, particularly in the legal sphere. Since the 1990’s, “neurolaw” has emerged as a new cross-disciplinary field of study. At the heart of heated debates lies the recurrent question of criminal responsibility, and an enthusiasm, just as recurrent, for an alleged overthrow of this notion by the fast-growing discipline of brain sciences. The theme of a neuro-revolution is indeed popular in the media and scientific and philosophical literature.

However, the link between science and law – between the explanatory and the normative – is far from self-evident, and the ties between neuroscience and criminal responsibility are still far from convincing. The alleged, and supposedly challenged, foundations of criminal responsibility (not least of which is the notion of free-will) are not only wrong. The real foundations of responsibility, embedded as they are in our daily experiences and ideological framework, are relatively impervious to scientific facts. They are susceptible to the latter, but only insofar as these may constitute an argument in favor of a political or ideological alternative. Moreover, the many limits (e.g., technical, interpretative, etc.) of neuroscientific tools and measurements call for increased vigilance when, if ever, using neuroscientific evidence in a courtroom.

Can neuroscience revolutionize criminal responsibility? Answering this question implicitly requires answering a second question: *should* neuroscience revolutionize the institution of criminal responsibility? Answering both, in turn, requires drawing the line between science and normativity, revolution and dialogue, fantasies and legitimate hopes. We aim here to introduce those nuances. In order to do so, we will first define criminal responsibility and elaborate on the principles and normativity behind this model. We will then address the limits to using neuroscience in the courts. Finally, we will evaluate the concrete and more modest contributions of neuroscience to the judicial process.

## What is Criminal Responsibility?

### Brief Definition and Basic Legal Principles

Before getting to the heart of the matter, some preliminary definitions are needed, especially regarding the definition of responsibility. As with any ambiguous term, “responsibility” allows for several meanings^1^: a tree falling on an electrical wire can be said to be responsible for a power failure (causal meaning), the captain of a ship is responsible for safety on board (role), a young man can be particularly irresponsible (character), insurers are responsible for compensating road accident victims (civil liability), a patient can be diagnosed irresponsible by psychiatrists (capacity), I can be responsible of my own misfortunes (authorship, or practical meaning), and so on. Criminal responsibility mixes different meanings (practical and capacity), but applies especially to social and legal norms (normative meaning). More specifically, a person is *prima facie* criminally responsible when he or she commits a crime while validating its constitutive elements: the *actus reus* and the *mens rea* (Box 1). The *actus reus* is the material element of a crime, which is to say the act that is being reprimanded, and the *mens rea* is the mental element, which is to say the state of mind of the accused at the moment of committing that act. A murder, for example, requires both the act of killing a person and the specific intent of killing that person. Without this *mens rea*, the act of killing someone does not amount to murder, but manslaughter. *Mens rea* is evaluated either subjectively through intention, carelessness or wilful blindness, or objectively, in comparison with a “reasonable person” facing similar circumstances, through negligence or recklessness. The elements required to prove those states of the mind are knowledge (of the nature of the act, of its consequences and of surrounding circumstances) and will (in the sense of a wilful act, i.e., an act that is part of a conscious plan of action). All of these terms have the same meaning as in ordinary language.

**Box 1. Criminal responsibility.**

Criminal responsibility is based on the *actus reus* and the *mens rea.* To be criminally liable, one must thus (1) consciously will to *x*; (2) know that *x* is wrong; and (3) do *x.* The presence of neurological prior causes to that action, or the predictability of an action due to identified priors, is a matter that relates to free will (how does one forms intentions? where do they come from? etc.). Responsibility, on the other hand, only cares for the feeling of consistency in the causal chain between intention and effect (*intention-action-effect* chain). What judges evaluate is the accused’s capacity to act in accordance with his or her intentions. The accused’s narrative on his or her agency is then normatively evaluated: that is, the narrative is confronted to current common beliefs and values. If you would report having intentionally killed your neighbor while knowing that it was wrong at the time you did it, but add that you did so following Satan’s orders, you would not be considered liable for your acts because you don’t share the Law’s normative reality: a secular reality in which Satan does not exist. Criminal responsibility, hence, lies in the individual’s subjective experience of agency and on the normative assessment of that experience.

To understand the scope of criminal responsibility, it is also important to grasp its limits, and hence, the classification of legal defenses. In Canadian criminal law, for example^2^, defenses are traditionally divided into two categories, and relate to situations affecting the capacity to orient one’s actions either “cleverly (intelligently)” or “freely”^3^. The first is composed of factors such as minor status, mental illness, automatism, intoxication, and error, while the latter includes necessity, coercion, provocation, impossibility, and self-defense. Briefly put, we excuse the incompetent: those who cannot understand or could not act. In ordinary language, this corresponds to the distinction between excuse and justification. An excuse is exculpatory because it casts doubt on the presence of *mens rea.* Justification, on the other hand, is a mitigating factor that reduces either the infraction or the sentence, since it intervenes after the *actus reus* and *mens rea* have been proven.

This definition of criminal responsibility outlines a particular vision of the responsible agent. *Mens rea*, as well as the typology of excuses, reflects the expectations we have of finding certain capacities in the ones we judge. In this regards, it can be said that criminal law is “capacitarian” (Vincent, 2010). Responsible agents are thus individuals capable of orienting their actions intentionally, consciously, and more or less rationally in a manner suitable to the normative framework in which they act. Besides, they must not be coerced into violating that framework.

All of those criteria are evaluated according to the individual’s behavior. The *causes* of behavior are not taken into consideration. In other words, a person is excused on the basis of an automatism, for example, whether caused by a physical or supernatural phenomenon.

### Why Free Will Does Not Matter

In this section, we take the liberty to venture into the question of free will, as it continues to haunt neuroscientific discourse on responsibility. However, it is essential to take this elusive question for what it is: a ghost – a dead specter that resurfaces when it is not properly put to rest.

That discourse considers the foundation of responsibility to be free will, taken in a general sense as meaning the possibility of “avoiding wrongdoing” or of “acting otherwise”^4^. The notion of determinism, as put forward by neuroscience, by reducing each of our actions to their neurological and unconscious causes, and therefore treating them as mere events rather than wilful actions, would appear to render the possibility of alternative outcomes illusory. Consequently, we would not be responsible, unless some other notion could be identified to salvage human agency and thus, responsibility itself.

Admittedly, this is a grossly simplistic definition of determinism. The reason for this approximation is the ongoing dissension over the central notion of causality between scientists and philosophers (e.g., Frisch, 2014, for a review). In the interest of being inclusive, we will therefore refer to *determinisms*. One should also notice that debates surrounding free will and determinisms are metaphysical, hence arguing within and opposing different ontologies. As we hope to show, there is no need for solving such metaphysical debates.

Having outlined these precautions, we can now turn to the debate on responsibility, which is distinct from free will and practical in nature. In other words, criminal responsibility is not founded in free will but on practical, subjective and political considerations.^5^ As such, it is impervious to any truth about determinisms.

First, determinisms alone, even if true, do not annihilate the feeling that I have of controlling my actions. Indeed, I always have the luxury of contradicting anyone’s predictions on my behavior (Searle, 1984). This “subjectivist” objection, defended by Searle and others (Chisholm, 1976; Baertschi, 2009), is not to be taken for an argument against determinisms. It is rather an argument in favor of our current concept of a responsible agent. Not only would this argument promulgate a wrong definition of determinism (Russell, 1912), but it mostly does not seek to address determinism at all. It does not matter whether or not my intentions, my feeling of control, my actions and their results are predetermined, or “caused,” by non-conscious antecedents – such as preparatory activity in subcortical and frontal motor areas (e.g., Cunnington et al., 2003). The argument consists of emphasizing that what does matter is the power to *associate* a conscious will to an act or to the results thereof. Therefore, our actions need only conform to our intentions and be perceived as part of a conscious plan of action, i.e., a plan that integrates an explicit and non-ambiguous representation of the action’s potential consequences (see Synofzik et al., 2008). The institution of responsibility thus lies in the possibility for the individual to experience *agency*, a subjective feeling of being causally responsible for his or her actions and consequences thereof (Haggard and Chambon, 2012) (see Box 2, “Sense of agency”). Furthermore, determinisms, even if they were proven to be true and deeply anchored in agents’ beliefs, do not modify the conscience about what is appropriate and what is not, what is deemed socially acceptable or unacceptable. Determinisms do not forbid the promulgation of norms. Knowing that I can always act in conformity with my intention and still tell apart right from wrong, I can more often than not decide to act rightfully, or at least believe that I am. In this respect, criminal responsibility relies mostly on our subjective experience, the impression of being able to choose to act or avoid acting.^6^

**Box 2. Sense of agency.**

Agency refers to an individual’s capacity to initiate and perform actions, and thus to bring about change, both in their own state, and in the state of the outside world (Chambon et al., 2014a). Thus, agency is an objective fact, demonstrated by individuals’ behaviors and the consequences of those behaviors. But agency has a first-component as well: it involves a subjective experience unique to the agent. The *experience* of agency, also referred to as “sense of agency,” is classically defined as a phenomenal experience of “mineness” of one’s own action (Synofzik et al., 2008; Eitam and Haggard, 2015). Whether this (minimal) action-related self-awareness relies on a *post hoc* cognitive *reconstruction*, or relies on internal signals being experienced while preparing and executing the action (e.g., Chambon et al., 2014b), is of little relevance for judging criminal responsibility. Criminal responsibility is acknowledged when, together with the material element of a crime (actus reus), criteria for *subjective* agency (whether the agent is sensing, experiencing, or reporting to have some sort of authorship over an action) are met.

Making a choice vs. Having a choice

Shepard and O’Grady criticize the univocal use of “choice” in folk psychology (Shepard and O’Grady, 2017). In a recent empirical work, they show that there are at least two distinct, though related, concepts of choice: one expressed in the phrase ‘making a choice’ and another expressed in the phrase ‘having a choice.’ “*One difference between these concepts,”* argue the authors, *“involves the kinds of alternatives each is sensitive to. Making a choice is primarily sensitive to whether or not psychologically open alternatives are present and whether an agent’s decision goes through normal psychological processes, but only minimally sensitive to whether or not genuinely open alternatives are present (…) In contrast, having a choice is sensitive to whether genuinely open alternatives are present, and whether psychologically open alternatives are present*”. Shepard and O’Grady relate this conceptual difference to a judgment on free will (which in turn they relate to responsibility). While they acknowledge that only few studies have investigated this link between choice and free will, with conflicting results, they note that “*findings suggest attributions of free will more closely mirror attributions of making a choice than having a choice*” (see also Shepard and Reuter, 2012; Nahmias and Thompson, 2014; Nahmias et al., 2014). The conceptual distinction between “having a choice” and “making a choice” echoes another distinction between causal responsibility and criminal responsibility, which we mentioned above. Thus, studies showing that the number and the availability of alternatives (*counterfactuals*) influence judgments on causal responsibility (Kulakova et al., 2017) are not directly relevant to determine what influences judgments of moral and criminal responsibility (Shaver, 2012).

Some legal and popular expressions may lead us to think that responsibility is nonetheless grounded in free will. Everyone legitimately assumes, for example, that criminal proceedings aim at evaluating if the accused “could have acted otherwise.” H.L.A. Hart, famous legal philosopher, takes the “fair chance of avoiding wrongdoing” to be the foundation of criminal responsibility. However, our previous paragraph has already shown the subjective interpretation plausibly given to this standard: the accused only needs to have had the impression of being able to avoid wrongdoing. The political interpretation of that standard shall now cast away free will once and for all. What matters most in the principle of a “fair chance of avoiding wrongdoing” is the word “fair” rather than “chance,” and the word “fair” taken as meaning equitable rather than just. It is not about knowing if there was actually a chance – another possible course of action – but rather if the circumstances as offered by society or a given social environment, were equitable and favorable to the expression of a singular “conscience” through a choice. By analyzing *mens rea*, the judge does not wish to know if the accused could have acted otherwise, but if the circumstances surrounding the crime were preventing awareness, and a sense of authorship, of the act.^7^ Society assesses the fairness of the conditions it gave to the accused that it is judging. Finally, this equity is based on the subjective belief that we consciously guide our actions according to our own motivations and on the collective belief that we are, in fact, endowed with motivations in the first place.

Anecdotally, it may be added that this observation is echoed in folk psychology. Recent studies have revealed the possibility that blaming might depend less on the availability of actual open alternatives than on the availability of psychologically open alternatives, i.e., that blame might be based on the appreciation of the accused’s subjective belief of having made a choice (Shepard and O’Grady, 2017). Those studies have demonstrated that there is a conceptual difference between “having a choice” and “making a choice,” and that it is possible that the second category is more relevant to the act of judging one’s responsibility (see Box 2, “Making a choice *vs*. Having a choice”).

One shall recall the great attention paid to Libet’s famous experiment and Wegner’s illusionism (Libet, 1999; Wegner, 2002). Following Libet’s results showing that a certain brain activity related to conscious actions systematically preceded the agent’s conscious intention, multiple interpretations suggested that conscious will was not the cause of our actions^8^, that we had no free will, and that we therefore could not possibly be responsible (Sinnott-Armstrong and Nadel, 2010). Without commenting on the validity of such theses (see Schurger et al., 2012; Frith and Haggard, 2018), it is obvious from our previous analysis that these do not impact criminal responsibility. They might have had an impact if criminal responsibility were based on free will (and in this case, more specifically, on the absence of neurological prior causes). However, as we have already pointed out, this is not the case. As long as the illusion of free will remains intact, even if it is an illusion, we can claim to be responsible. The responsible agent is only required to have an internal plan of action, including a representation of the planned behavior (intention), and to have sufficient insight into the normally possible consequences of that behavior (knowledge) (Synofzik et al., 2008). In this regard, the origins of an intention do not matter. What criminal responsibility requires is an individual’s capacity to act in a manner deemed appropriate to the realization of the related intention, given his or her knowledge of social norms defining what is acceptable and unacceptable.

## Understanding Normativity: Neuroscience Tells but Does Not Compel

Responsibility is immune from determinisms not only by virtue of its independence from free will. In fact, no scientific discovery, as significant as it may be, in and of itself calls for the overthrow or modification of a social institution. In other words, we insist on the difference between positive and normative, also called the ‘is-ought gap,’ and will explain further the particularities of normativity.

### The Morse Challenge

Hume brought the irreducibility of *what is* to *what should be* to light in the XVIII century. The idea goes as follows: nothing that simply is calls directly for what should be, *without postulating that “what should be” (what is good) should be conform to what is.* At the junction of law and neuroscience, S. J. Morse reaffirmed the Humean argument to defeat naively enthusiastic scientific claims in courtrooms. In his famous article “*Brain overclaim syndrome and responsibility: a diagnostic note*” (Morse, 2006), he recalls the behavioral, as opposed to cerebral, criteria for responsibility and insists on the incapacity of brain imaging to set the threshold of normality vs. abnormality either in ethics or in law. “*Brains are not responsible. Acting people are*” (p. 406)^9^. Hence, explaining the difference in behaviors between a teenager and an adult by the lack of complete myelinization of cortical neurons as in Roper v. Simmons (2005)^10^, and inferring as a result the lack of sufficient responsibility to qualify for the death penalty, is simply irrelevant (p. 397).^11^ It only takes a difference in behavior between those two types of individuals. Baerstchi complements the “Morse challenge” by showing concrete Humean limits in some experiments (Baertschi, 2009). Some studies have outlined the different brain areas which operate in the course of moral decision-making, when faced with the well-known trolley dilemma (Roskies, 2004). Those areas, while of interest to indicate the part played by emotions in moral decisions, do not inform the manner, consequentialist or deontologist, in which to settle this dilemma.

### Responsibility Is a Normative Concept

The requirements for responsibility are normative, which is to say that they are standards that claim to originate in a social choice and to have practical authority. These norms are guided by beliefs and principles.

For example, the legal principle of non-retroactivity: according to this principle, it is fair to be judged only by laws that you had the opportunity to know about before committing an offense. This principle implies that individuals are capable, or believe themselves to be capable, of orienting their actions so as to avoid negative consequences (here, a criminal penalty). One way to take this principle and the belief it implies into account is to establish the state of mind of the accused at the time of the events. Considering, as highlighted above, that *mens rea* also serves the purpose of ensuring the equity of the circumstances in which the accused acted and thus ensure that he or she had a “fair chance of avoiding wrongdoing.”

The responsible agent’s abilities, such as intentionality and rationality, are also normative. Phineas Gage is a classical example. In this case, a man who suffered great brain lesions after an accident started to adopt negative behaviors. When thinking abstractly, he could make a good decision, but, when facing a concrete situation, he would systematically make a bad one. However, when deeming his behavior as good or bad, we already interpret his actions according to a normative standard of rationality (Baertschi, 2009). Gage was incapable of reasoning about a decision directly related to him or his personal circle, of acting rationally according to his best interest (whatever definition of interest is taken). We then consider that he lacks an essential characteristic of practical rationality, i.e., the ability to apply logical reasoning to a concrete objective deemed beneficial. Once more, this conclusion relies on a common definition of rationality and does not rely on Gage’s brain injury.

Another example of normativity at work in responsibility assignments concerns “reality” itself. For some God exists, for others he does not. Depending on whether we are atheists or believers, “God has asked me to do it” is either a madman’s whim or a saint’s word. The difference between the madman and the saint is not so much a question of belief than it is a question of norms and society. The madman is a saint if we share his reality, and the saint a madman if we don’t. An implicit norm is thus at work in any legal judgment, as minimally relating to reality. Our beliefs are involved in what we deem rational. What we recognize to be rational is partly arbitrary, precisely because we recognize it.

In the previous section, we insisted on the experiential requirement: the accused must be able to report a feeling of agency to potentially be responsible. We added in this section another criterion: responsibility also depends on a normative appreciation of that subjective experience, i.e., a normative attribution of agency (of what we commonly call agency).

### Changing the Premises of Responsibility Is a Social Decision

To be efficient at an institutional level and in order to inform juridical considerations, neuroscience must accept that scientific facts alone are not enough, and that these must be integrated into a broader normative scheme if they are to have any legal significance. It must convince us beyond and against our daily experiences that our rationality is sufficiently flawed, that our will is powerless, that our choices are all about neurological prior causes, to the point that we should doubt everything we are told by this “rationality,” this “will” and those “choices,” etc. It must acquire normative authority. After all, why not? Ancient Greeks certainly did not have the same individualistic appreciation of the artist’s agency: the writer would simply copy words dictated by muses. Neuroscience would nonetheless be leaving the field of science for the bumpier grounds of ethics and politics. They would then have to face the obvious: in terms of normativity, truth is on the side of folklore. The common intuition about our agency reverses the onus of proof: it’s up to neuroscience to convince us that we don’t have it.

Finally, we would like to present a few arguments in favor of resisting a potential neuro-conversion of criminal justice policies. We have already discussed the logical impossibility to go from positivity to normativity without additionally postulating that “what should be should conform to what is.” This postulate, however, needs further elucidation.

Taken in a broad interpretation, this premise actually translates into a principle dear to justice: “no one is expected to do the impossible.” To be fair, we can only ask of ourselves things that we can achieve. According to this principle, one might think that neuroscience is better suited to establish a basis for responsibility since, by definition, they would only require what is accessible to human nature. However, this would be forgetting that law does not ask for perfection. To a certain extent, law is meant for the humans we are. When judging an individual’s rationality, legal reasoning only takes common standards and expects an average fulfillment thereof. Neuroscience would thus not be fairer than law is in this regard.

The strict interpretation of the premise, i.e., the claim that description should translates into prescription (for example, taking the cortex myelination of teenagers as indicative of their lack of liability), weakens the law rather than consolidating it. Indexing normative standards to the current state of science dooms the latter to follow the vagaries of a branch of science that is necessarily evolving, often imperfect, sometimes flat out wrong while consensus arises and disputes settle. One notice in this respect the fast development of paradigms in cognitive sciences (from phrenology in the XIX century, to radical behaviorism in the 1930’s, cognitivism in the 1950’s, enactivism in the 1980’s, etc.), and the legal incongruities that would arise from following such paradigms. This would lead to legal instability that goes against some fundamental principles of justice such as the necessity of having an explicitly enunciated law beforehand^12^. Past and current law, based partly on general criteria inspired from daily experiences, showcases continuity and stability, which science could not guarantee.

Moreover, the strict interpretation of the premise ignores a second principle dear to any normative framework, i.e., the principle of perfectibility. “Principle” might be too strong a term, and some might prefer using “aim.” All things considered, perfectibility is a truism of normativity. A normative framework, while restrained to accessible requirements, still posits those requirements as desirable objectives to aim at. Those requirements can be mediocre, but everyone must at least aspire to mediocrity. The vision of a perfectible individual would be missing in a framework that ignores this aspiration. Such a framework would freeze men and women in their identified and limited abilities, without being able to legitimate the expectation that they give the best of themselves.

## The Limits of Neuroscience

The previous paragraphs should not be read as ignoring the law’s own flaws and limitations. Classical criticism of behavioral requirements for criminal responsibility points to the risk of circularity inherent to behavioral evidence, especially in assessing mental disorders: the absence of responsibility for antisocial acts would be assigned due to a mental disorder whose main, if not only, symptoms are those very same antisocial acts. That particular criticism has been amply discussed in the 20th century in a notorious debate opposing Lady Barbara Wootton and H.L.A. Hart (Matravers and Cocoru, 2014). Wootton supported that in *R v. Byrne*, “*the extent of Byrne’s depravity was itself taken as evidence of his lack of responsibility*” (Wootton, 1963)^13^. While Hart nuanced that claim by reiterating the importance of circumstantial evidence at the time of the events in evaluating *mens rea*, the distinction between mad and bad remains a delicate one. In itself, a wrongful act does not sufficiently evidence the incapability of distinguishing between right and wrong, although the former is indeed a probable consequence of the latter. In the same vein, the more evil exceeds a reasonable person’s imagination, the more it is associated with a deficient reason. Neuroscience might then be useful to the law. It could confirm or invalidate behavioral evidence. Besides, it already has been used in courts (see next section). However, precautions are once more in order. Neuroscientific evidence is restricted by technical and legal limits. We identify them here.

### Technical Limitations

These limits are already addressed extensively in the literature (e.g., Pardo and Patterson, 2013; Kedia et al., 2017; Haushalter, 2018; Pardo, 2018). We will simply enumerate and describe them briefly:

#### Temporal Limitation

Neuroscience and its tools – especially brain imaging – can only prove permanent anomalies, still visible at trial, and not temporary conditions concurrent to the time of events and already dissipated at trial. Moreover, it is impossible to know whether the anomaly observed is anterior or posterior to the crime (Vincent, 2010, p. 95). Finally, as highlighted by others (Maibom, 2008; Reimer, 2008; Vincent, 2011), the permanent condition must also be linked to an inability to be responsible (i.e., an inability that paralyzes judgment) and not simply to a general feature of the accused’s character, such as aggressiveness.

#### Interpretative Limitation

A first limit relates to the interpretation of functional imaging data (e.g., fMRI) and the risk of evidential circularity. Without diving deeply into the philosophical debate around mental states multiple realizability (e.g., Aizawa, 2009), it remains difficult to accurately map a cognitive process or function in a precise brain area, neural network or population. This difficulty arises from the fact that one brain area can perform different functions (*many-to-one* mapping) that are hardly distinguishable without an appropriate experimental protocol. Partially overlapping activity patterns associated with distinctive functions also complicates the proper interpretation of brain scans when they are not concurrently read with the patient’s behavior (for example, when neural circuits required for an action’s execution partially overlap with some linked to the observation of that same action executed by a third party, if not with the simple imagination of that action, see Jeannerod, 2001, for a review). The necessity of always going back to the behavior to interpret a functional scan makes brain activity evidence circular: it is used to prove or explain a behavior, and yet, brain activity patterns only mean something in so far as they are associated with the behavior they seek to explain (see infra, our criticism of P300-MERMER; see also Krakauer et al., 2017, emphasizing the better epistemological accuracy of *behaviourally driven* neuroscience). Hence, exclusive neural evidence, just as strictly behavioral evidence, does not solve Wootton’s circularity issue mentioned above. Again, looking at the circumstances surrounding the alleged crime is necessary. Because brain scans are rarely informative in themselves – without referring to the behavior they seek to explain – there are few situations in which they are useful for establishing criminal liability. They may only be in distinguishing the truth in “gray area” cases “in which the behavioral evidence is unclear” (see Morse, 2019)^14^.

A second linked limit is the risk of producing reverse inferences (see Poldrack, 2011), i.e., inferring a mental process from the observation of activity patterns without consideration for the actual behavior or the circumstances thereof. Reverse inferences can lead to fallacious interpretations of neuroimaging data such as: concluding that a blind woman sees because her visual cortex activates; or coming to the conclusion that dogs understand words of praise because some patterns, as revealed by fMRI, activate in their left brain hemisphere (Andics et al., 2016)^15^. It is worth noting that reverse inferences are often wrongly used as a common strategy to interpret experiment results. The problem is that neuroscience still does not have a sufficient understanding of brain functions to infer mental process on the sole basis of neural activity^16^ (for a similar critic see Kedia et al., 2017). Reverse inferences, although tolerated in the context of exploratory scientific practices, is thus not fit for law’s requirements, in particular considering the institution of criminal responsibility and the major consequences it brings about for an incriminated individual.

Let us note that this critique also targets the most recent tools used for probing neural activity, including brain data decoding techniques based on machine-learning (e.g., Multi-Voxel Pattern Analysis). The thesis that referring to behavior is essential to the correct interpretation of brain activity grows in importance when applying data-driven methods to decode the accused’s intentions or thoughts. Indeed, nothing in “decoded” activity patterns alone indicates whether the brain is actually using those patterns to complete a task or to achieve a specific cognitive goal. In other words, it is still necessary to show that the pattern decoded by machine learning algorithms actually contributes to the studied behavior. This requires being able to explicitly link decoded patterns to behavioral outputs (e.g., Ritchie and Carlson, 2016). Without an explicit reference to behavior, decoded activity patterns have but weak explicative value: the possibility always remains that they might only reflect associative processes concomitant to the relevant functional process, e.g., the reuse of sensory information for higher-level operations (Ritchie et al., 2017; Bouton et al., 2018, for a review).^17^

#### Comparative Limitation

To be significant, fMRI scan results must be replicable and subjected to group analysis. An fMRI scan is a functional scan that measures and maps brain’s activity while the subject is completing a task (e.g., encoding information, storing it, using it to make or guide decisions, etc.). Specifically, what is measured is an indirect effect of brain activity, i.e., a modification of oxygen levels in local blood supplies (blood-oxygen-level-dependent response, or BOLD signal). This measurement is considered as a reliable indicium of a specific brain area being required to do a task, if not essentially “doing” that task. However, linking BOLD signal variations to cognitive processes remains difficult for three reasons: (1) even in a resting state, the brain presents spontaneous activity fluctuations; (2) neural computations have intrinsic noise; (3) what one does or what one thinks in a scan can never be completely controlled. It is thus imperative, before introducing fMRI scans in courtrooms, to conceive experiments carefully designed to isolate, in an individual’s brain, activity fluctuations relevant to the behavior being studied, i.e., experiments (factorial or parametric designs) that discriminate between relevant neural activity and background or task-unrelated neural activity.

In this regard, Kedia et al. (2017) recall the importance of replication and generalization in order to assess fMRI measurement reliability. These require a great number of observations/acquisitions in the view of minimizing the signal-noise ratio, as well as replicating results between individuals or cohort in order to avoid statistical artifacts. Accordingly, the interpretation of functional scans from a single person (for example, the accused in a trial) is extremely dubious as it is vulnerable to type I (false positive) and II (false negative) statistical errors that can only be avoided through robust group analysis and rigorous experimental protocols.

#### Normative Limitation

The relevance of results, be they from functional or anatomical scans, depends on the (normative) definition of handicap linked to a certain behavior. For example, anatomical scans (the equivalent of pictures of the brain structure) can reveal anatomical alterations and anomalies (e.g., loss of cerebral matter, alteration in the organic structure, excessive spinal fluid, etc.). Relevantly producing such evidence, however, implies the hypothesis that those anomalies alter the accused’s capacity to follow or detect a norm, or to adapt to or adopt an appropriate behavior. Anatomical anomalies alone do not indicate the presence of a handicap, and do not necessarily translate into mental deficiencies. Extreme examples exist of people having one entire hemisphere removed (hemispherectomy) and yet, not experiencing any abnormal difficulty in their daily lives, even when the hemispherectomy has been performed at a late stage of development (Schmeiser et al., 2017)^18^.

A functional or anatomical anomaly is interpreted as being a handicap only insofar as the behavior it might produce is considered such. To say that a subject is not able to follow the rules due to brain injuries requires proving that these injuries are the source of that disability (as indeed, most penal codes prescribe). Neuroscientific tools may thus indicate the source of a disability (and not be the evidence of the disability itself). Yet, although some scientific findings prove that some prefrontal injuries generate sociopathic tendencies (e.g., Phineas Gage), not all prefrontal lesions lead to such tendencies. Structure–function mapping is, in fact, relatively flexible. Further, the brain is functionally vicarious: under certain conditions, new functions can emerge via the reuse, the recycling, or the reconfiguration of existing brain circuitry (e.g., Anderson, 2010; Wittenberg, 2010). Interpreting functional or anatomical anomalies remains questionable, and cannot forgo referring to the abnormal subject’s behavior.

#### Experimental Limitation

Laboratory conditions and actions typically tested do not necessarily reflect the conditions of daily life in which individuals normally act (see Box 3). Participants’ movements, for example, are extremely restricted in a scanner (any head movement superior to a few millimeters can jeopardize results and produce false positives^19^). Experiments testing an agent’s intention, choice and responsibility are more exposed to this line of criticism. Some have argued that the actions participants are asked to perform (such as pressing on buttons or targets, or following a sequence of buttons pressed following an audio signal, etc.) are not intentional since they are not chosen. More precisely, they are triggered by exterior conditions/demands and they are almost automatic, without any surprise and spontaneity as to the when and how (Brass and Haggard, 2008; Waller, 2012). Furthermore, A. R. Mele shows that what is called “intentional” varies from scientists to philosophers, and that some actions can be considered as intentional even when following strict instructions or when not being fully conscious (Mele, 2009; Chambon et al., 2011; see also Pacherie, 2008, for a three-tiered dynamic model of intention). Despite this nuance, it is obvious that “*the arbitrary free choice afforded participants in the experiments, the choice of when or whether to perform a simple movement, is disconnected from participants’ everyday justificatory or motivational reasons—moral, prudential, or otherwise—for action and thus fails to capture the type of decisions and actions for which agents are typically held morally responsible”* (Waller, 2012)^20^. Neuroscience could nonetheless compensate for this shortcoming through revisited protocols.

**Box 3. Representativeness of fMRI participants.**

The representativeness of fMRI participants has been questioned. For example, people who do not meet inclusion criteria for fMRI scanning are automatically excluded from neuroimaging studies, including individuals wearing tattoos or permanent jewelry, devices or metal in their body (whether aneurysm clip, pacemaker, or metal fragments), pregnant women, etc. Also, most neuroimaging data are collected from student subjects pool, and from Western, Educated, Industrialized, Rich, and Democratic (WEIRD) populations more broadly (on WEIRD people, see Henrich et al., 2010; see also Baumard and Sperber, 2010 on WEIRD experiments). These samples may differ in many concrete ways from broader populations of interest (Falk et al., 2013). Early life experiences are rarely taken into account when screening and recruiting participants; yet parenting and socio-economic status (SES) have effects on brain areas such as the amygdala and prefrontal cortex, whose dysfunction has been linked to a variety of legally relevant outcomes such as crime and violence, drug use, and reduced cognitive control (see Falk et al., 2013, for a review)

Statistical reliability of fMRI results

The reliability of neuroimaging results has been the subject of much discussion (for a review, see Eklund et al., 2018). Various software used in fMRI analysis have bugs that increase the rate of false positives, i.e., the probability of finding a significant activation (yet a statistical artifact) in a specific region during a given task. In a recent paper, Eklund and colleagues estimated that about 10% of the fMRI experiments in the literature – thousands of fMRI studies – were in doubt and could have produced at least one false positive. It is possible to control the false-positive rate in fMRI by correcting from multiple comparison, a gold standard of statistical massively univariate analyses such as fMRI. However, the type of correction that should be used is also a matter of discussion (e.g., Woo et al., 2014). Indeed, an appropriate balance must be found between trying to minimize false positives (Type I error) while not being too stringent and omitting true effects (Type II error) (Han and Glenn, 2018).

Ecological validity of fMRI experiments

Serious doubts have been raised as to the admissibility of fMRI evidence in judicial settings. Due to their lack of ecological validity, neuroimaging studies – laboratory experiments in general – can prompt behaviors that have no real functional meaning but in the constrained space of the scanner. This could be the case of the so-called “altruistic punishment” behavior, whereby individuals “punish” defectors or free-riders although the punishment is costly for them and yields no material gain (Fehr and Gächter, 2002). However, what is observed in natural settings draws a different picture: individuals who are identified as free-riders are generally not “punished” but either ignored or simply excluded from any subsequent transactions in favor of other, and potentially fairer, partners. In other terms, laboratory volunteers would engage in altruistic punishment because, in the reduced space of the experimental room, they would not be given “outside options,” e.g., the opportunity to find more cooperative partners (Guala, 2012; see also Barclay and Raihani, 2016). This observation echoes a recent study showing that people punish altruistically because the experimental setup (an economic game with oriented instruction) incites them to do so – a phenomenon known as “experimental demand” (Pedersen et al., 2018).

As a final remark, it is worth pointing out that neuroimaging can (and will undoubtedly) contribute to make the assessments of criminal liability more objective than other – and sometimes more idiosyncratic – behavioral assessment tools within the traditional context of criminal law. While saying this, we must also recall that law’s criteria are first and foremost behavioral – actions and mental states are what are judged. Thus, while we recognize that classical behavioral assessments can be distorted by the expert’s subjectivity, it should also be noted that behavioral data can readily be translated into notions that speak the language of law, while neural data are rarely self-explanatory, especially not with respect to the defendant’s behavior (see above for the evidential circularity of functional neuroimaging evidence).

### Legal Limitations

Legal limitations might be more severe than technical limitations since overcoming them depends on exclusively legal debates. However, they inform neuroscientists who wish to assist the courts or to simply legally contextualize their scientific findings.

First, neuroscience can only impact legal excuses and not legal justifications. By definition, the latter concerns external restrictions to an agent’s actions. An agent’s actions will be justified due to the existence of only one reasonable solution to a problematic situation. Arguments relating to neurological conditions reducing possible options (such as “my brain was in such a state that it was impossible to avoid acting a particular way” or “my brain did it, not I”) do not intervene at this stage. Justifications do not only tackle phenomena out of will power’s reach (like electrical pulses in neural circuits), but precisely phenomena completely independent and external to the agent, including its neural circuits. Justifications are about circumstances external to oneself, or even actually contrary to oneself since all the goodwill in the world could not prevent wrongdoing. This is the case with self-defense, for example, when circumstances someone faces only allow for two options - kill or be killed-, knowing that the latter option constitutes the threshold beyond which obedience becomes illegitimate.

“Impossibility” is also a corresponding line of defense. Its definition in Canadian law is precisely “an exterior, unpredictable and irresistible cause that prevents the individual, despite his or her own will, from conforming to the law”(Parent, 2008, p. 769)^21^. “Necessity” is another legal justification that follows the same rationale, although more flexible as it allows the possibility of choosing between two evils. Aristotle notoriously illustrated the situation of a mixed act (intentional but constrained) through the story of a captain’s ship.^22^ Moreover the standard of appreciation of all those justificatory factors is objective, which means that it applies the standard of “the reasonable person” placed under the same circumstances (Parent, 2008). Objective evaluation in these cases serves the purpose of knowing whether or not the alleged crime was bound to happen independently from the accused’s personal characteristics. In this regard, scientists should pay particular attention not to comment on legal justifications when addressing the issue of criminal responsibility^23^.

Finally, any evidence submitted at trial, be it scientific or not, has to be validated by certain legal tests before being accepted and presented to a jury. These tests generally ensure that the accused’s rights and the constitutionality of investigating methods are respected. They allow, for example, excluding evidence (even if overwhelming) that would come from an unlawful search in the accused’s house. A similar degree of vigilance applies to technical evidence, such as expert testimony, medical reports, etc. In American law, for example, evidence must be admissible and relevant.^24^ One of the criteria for admissibility, as elaborated in Frye v. United States (1923) and known as the Frye Test, is the general recognition of the evidence’s experimental value by the appropriate scientific community (see Box 3). While adopted just under a century ago, the Fye Test still serves today to exclude non-consensual techniques, e.g., to restrict the use of genetic evidence of behaviors in federal habeas corpus cases (Cullen v. Pinholster, 2011; Kaufmann, 2013). The Daubert trilogy in 2002 then clarified and modified provision 702 ruling over testimonies and expert reports^25^. The Daubert Test establishes the following admissibility conditions: (1) the expert report must be based on sufficient facts and data; (2) the testimony is based on reliable principles and methods; and (3) those principles and methods have been faithfully applied to the facts in question. Those criteria are, however, neither exhaustive nor exclusive, and others have been developed: whether the evidence submitted belongs to the expert’s usual field of research or on the contrary have been elaborated in anticipation of the trial (Daubert v. Merrell Dow Pharmaceuticals, Inc, 1995) the consideration of alternative interpretations, (Claar v. Burlington, 1994) the influence a lucrative contract might have exercised over the expert’s diligence (Sheehan, 1997), the general reliability of the expert’s field of study Kumho Tire Co. v. Carmichael (1999) the presence of extrapolations in the expert’s reasoning, General Elec, Co. v. Joiner (1997) and others.^26^ The more recent case of Terry Harrington v. State (2003) set even clearer and more concise admissibility criteria: (1) the previous publication of the submitted tests and methods in blind-peer reviewed journals, (2) the testing of these methods outside laboratory in real life conditions, and (3) scientific community’s approval thereof (Frye Test) (Pallarés-Dominguez and Esteban, 2016) (see Box 3). Besides being admissible, evidence must also be relevant pursuant to provision 403 Federal Rules of Evidence. It must be highlighted as well that these tests, although similarly and generally requiring admissibility and relevance, vary from one jurisdiction, country and legal tradition to another.^27^

Now that leeway for neuroscience has been defined, we can look into concrete attempts at introducing such techniques into courtrooms.

### Lie Detectors

A P-300 MERMER test (Memory and Encoding Related Multifaceted Electroencephalographic Response) or *Dr. Farwell’s brain fingerprinting* (e.g., Farwell and Smith, 2001) is not exactly a lie detector. Rather, it highlights the accused’s memory, or absence thereof, about certain facts, by measuring a positive brain wave called P300 MERMER. A certain wave potential obtained through relevant stimulus would show the presence of an actual memory linked to this stimulus. Proponents of this technique measure the wave amplitude from P300 responses to images or words linked to familiar events or events recognized by the accused: a crime, terrorist training, bomb-crafting knowledge, etc. The test produces a neural signature for the absence or presence of relevant information in the accused’s memory, and gives a reliability index for that result. Experiments in and outside the laboratory have shown an error ratio of less than 1% (Farwell, 2012, for a review). P-300 MERMER test has been used in a somewhat contradictory manner in the courts: in Harrington v. State (2003) it allowed for the release of a man wrongly convicted of murder after 23 years of imprisonment. However, in *State v. Grinder*, it has been recognized as a highly probative and incriminating evidence (Harrington v. State, 2003; Brandom, 2015). Other techniques have been developed, such as a TMS (transcranial magnetic stimulation) procedure that disrupts brain areas supposedly implicated in intentional trickery (e.g., George et al., 2006; Rosen, 2007), but they present less reliable results.

Those methods are questionable on many levels. Conceptually speaking, they contribute to “mereological fallacy,” that is the general tendency of neuroscience to ascribe to the brain, or parts thereof, abilities or properties that in fact belong to individuals. It wrongfully attributes a property of the whole to one particular mechanism (Pardo and Patterson, 2013). However, this conceptual objection is not consensual (Levy, 2014). In the same vein, the possibility of detecting lies is contested by the mere context-dependant definition of lying: “*As Don Fallis notes in an insightful article, the difference that makes “I am the Prince of Denmark” a lie when told at a dinner party but not a lie when told on stage at a play are the norms of conversation in effect*” (Pardo and Patterson, 2013). A false declaration is thus not always a lie and depends on whether or not it is stated in a conversational context whose norm is “you shall not make false declarations.” Nevertheless, participants in lie detecting experiments are precisely instructed to utter false declarations, and therefore perform in a context antithetical to lying. Besides, someone can lie without knowing it, when stating something false and yet believing it is true (Faulkner, 2007). One can also convince oneself that a false information is in fact true (Van Horne, 1981; see also Pardo, 2018, for a critical review). In other words, what neuroscientific tools record are not lies. On a more practical note, some authors worry that it would already be possible to elaborate counter-measures in order to cheat lie detectors (Kedia et al., 2017).

On a strictly technical level, P-300 MERMER test results are more than doubtful. Most of the studies on which that method rely, focus on small biased samples (often student volunteers rather than real accused in real investigation conditions). Most of the studies on the accuracy of that method are rarely reviewed on a blind-peer basis.^28^ Moreover, the 20 fingerprint standards defined by Farewell himself to evaluate his own method’s efficiency are controversial. Some scientists consider them to be purely subjective and self-confirmative, as they are not defined by a scientific consensus (Meijer et al., 2013). One of the most severe criticisms comes from one of Farewell’s mentor (Dr. Donchin), who criticized the (laboratory) conditions in which it has been mainly tested. In real life conditions, some parameters must still be addressed: the reliability and efficiency of the electrophysiological response for real accused persons, for example, or neurologically atypical individuals. In sum, given the large differences between the typical experimental setting and realistic criminal investigations, it is questionable whether the results of P300 MERMER experiments can be generalized.

The relative impossibility of replicating Farewell’s method for independent researchers, at least with similar statistical power, should also be noted. Indeed when replications take place, results show less statistical strength compared to the original studies (88% of correct detections (Meijer et al., 2014), which is similar to results obtained through other techniques linked to the autonomous nervous system (Skin Conductance Response, Respiration Line length, Changes in heart rate, etc.). P300 MERMER’s accuracy is also vulnerable to counter-measures (see Rosenfeld, 2005, for a comprehensive review). It actually collapses when crime-based items are compared to irrelevant items with the largest P300 responses (Lukács et al., 2016). Finally, the test lacks sufficiently reliable baseline measures, that is: truly neutral questions asked to participants.

Mnemonic recognition of familiar details of events is at the heart of P300 test. This mnemonic recognition is nevertheless challenged by other limitations: the fact of having crime-based information stored in memory is not sufficient to infer guilt, as frequent or significant details for a participant might trigger that very same event-related potential; P300 is susceptible to false memories^29^ and also to lack of participant attention (“many guilty suspects ended up passing the test simply because they hadn’t paid attention to the objects in the test,” see Meijer et al., 2014). Finally, some have gone to the extent of questioning the whole of P300’s relevance and argued that the test’s benefit lies only in the examination strategy used (Classification Concealed Information, CIT) and not in the electrophysiological signal itself.^30^

One last legal objection is possible. P300 MERMER might indeed violate the right against self-incrimination.^31^ This right is one of the fundamental rights of the accused, namely the right to silence, the presumption of innocence (which shifts the onus to the Prosecution to prove allegations beyond reasonable doubt) and right to not be compelled to give evidence at one’s own trial, etc. In the case of Antonio Losilla, the argument was raised in court to appeal the decision authorizing P-300 MERMER (Lukács et al., 2016). Nevertheless, the test had been used before the decision was rendered. In the United States, *Schmerber v. California* found that the 5^th^ Amendment protected the accused from being forced into “*prov[ing] a charge from his own mouth*” but that it did not apply to material and physical evidence. That distinction between verbal and physical testimony has since been roundly criticized by jurists for its inconsistency with the objective of the right against self-incrimination (Farahany, 2012).

The main objections are conceptual, technical, and legal, and although each is limited in its own scope, together they seriously bring into question the use and rigor of such methods.

## No Revolution, Only Dialogues

Neuroscience’s claims relating to law generally can be separated into three categories: (i) revision or reform, according to which neuroscience overthrows current legal criminal standards; (ii) *evaluation*, which consists of using neuroscientific tools to play a role in the judicial process; and (iii) *intervention*, which translates into the direct manipulation of people’s brains (this clever classification is borrowed to Meynen, 2014). We have already established through Section “What Is Criminal Responsibility?” and Section “Understanding Normativity: Neuroscience Tells but Does Not Compel” that revisionist claims have no foundations. While keeping in mind the limitations addressed in Section “The Limits of Neuroscience,” we would now like to focus on cases suggested by the other two remaining categories, and will deal with several attempts at introducing neuroscientific elements in courtrooms.

### Irresistible Urges and Rationalism

Criminal law generally adopts an intellectualist/rationalist approach (as opposed to volitionist/will oriented approach) in evaluating an agent’s capacities. That is, it seeks to determine whether or not an accused has a functioning sense of reason, and not to assess the strength of his or her will. It thus recognizes deficiencies of rationality but not weakness of will. In Canadian law, for example, provocation is a defense that reduces murder to involuntary homicide due to a violent anger provoked by “an action or an insult of such a nature as to be sufficient to deprive an ordinary person of the power of self-control”^32^. It relies on the evidence of a momentary lapse in judgment and not a simple urge (see also Box 4). The expression “self-control” (and loss thereof) are not controversial and are associated with a “temporary suspension of reason” or “*the temporary eclipse of reason by passion as the guiding force influencing one’s action*”(R c. Gibson, 2001). The same rationalist approach applies to other behavioral disorders, such as pyromania and kleptomania. Being a kleptomaniac is not sufficient grounds for being exonerated from stealing because criminal law considers that a kleptomaniac still knows that what he or she is doing and that stealing is wrong. Some debates still shake the legal and philosophical community as to the validity of pleading irresistible urges and the voluntary aspect of acts, but rationalism prevails (Morse, 2002; Parent, 2008, p. 859).

**Box 4. Criminal responsibility under influence.**

Some concerns accompany the growing use of invasive technologies such as neural implants and “deep brain stimulation” (DBS) neurosurgical procedures. Patients having received DBS as treatment may exhibit various side effects, from developing new musical preferences to suffering from temporary hallucinations. What about cases where the implant would be the cause of a criminally blameworthy behaviour? Do these brain devices entail a revision of our legal categories about responsibility, just like assisted reproductive techniques have changed the legal definition of a parent?

Once again, the current law already has tools to address situations of potential concern caused by DBS. If the accused at the time of events perceives the reality differently from what it is (hallucination), she/he cannot be held responsible. The expert evidence about the role of the implant in the false perception would not be in and of itself exculpatory, but it would add to the credibility of the defence’s narrative. More generally, the law recognizes *intoxication* as a defence, as a state or an external influence that alters the accused’s perception and personality. *Voluntary intoxication* – drug and alcohol abuse – is not exculpatory because it is assumed that the accused knew about the adverse effects of the substance beforehand.

*Involuntary intoxication* (which may correspond to the side effects of a medication) is recognized as a valid defence. Then the question would be to know whether the potential adverse effects of DBS could be assimilated to involuntary intoxication. Thus, it would be possible to modify, and even rename, an already existing defence – involuntary intoxication – to include new interfering influences. However, this “new” defence would follow the same logic as the previous one. Once again, brain technologies do not revolutionize law but improve and marginally modify existing legal resources (see Klaming and Haselager, 2013).

In a similar vein, liability issues concerning the releasing of risky technology into the market are not a novelty. Our case could be compared to the pacemaker in this regard. The law already addresses many aspects of this issue (the patient’s consent, knowledge of the risks, transparency concerning the risks, professional insurance for doctors, etc.). We wish not to speculate about the wording of future provisions to deal with DBS but rather to stress that the law is already well equipped to deal with seemingly new objects.

Neuroscience would here claim that some behaviors that we take to be malevolent urges are in fact deficiencies of reason.

One of these claims relate to drug addiction. Neil Levy hence contends that drug addiction is not to be considered a compulsive behavior but rather to as altering judgment capacity: “*though most of the time addicts judge that they ought to refrain, at the time of consumption they judge that all things considered they ought to consume”* (Levy, 2014). This alleged contradiction would show that drug addicts suffer not only from a shortcoming of will power but also a disorder of reasoning. Moreover, drug addicts’ endorsement of their own behavior is equivocal. Neuroscience would then be more suited than behavioral evidence to establish that link, and could as a result lead to a not-guilty verdict or a verdict reflecting a diminished degree of responsibility.^33^

However, we can object that numerous drug addicts report knowing that what they do is wrong. They do not showcase a troubled reason that would not dissociate right from wrong. Levy argues that it is possible to be wrong about one’s own mental state and that subjective experiences can thus be erroneous (similarly to cases of erroneous affective attribution or cognitive dissonance). We cannot admit this answer: the flaw in a subjective experience is relative to a context and to an external observer, not to a neurological state. In other words, a drug addict who is acting illegally while cognizant that he or she is acting as such has no rational deficiencies. An external observer can only but note that action, thought and reality are all in agreement. We broadly consider that a person claiming to see Satan is mad because this subjective experience does not correspond to reality (again, the normative reality of a secular law that does not acknowledge Satan’s existence). What is deemed a bad judgment is normatively qualified from the outside. Cognitive disorders are disorders for the experts observing them. The subjectivist objection that calls for considering the subjective experience of drug addicts is thus valid.

The case of drug addicts reporting thinking that, at the moment they act, they are acting as they should, still needs to be addressed. Levy’s argument here takes advantage of the ambiguity of terms like “should/right/duty.” If science can show that drug addicts think that they are doing the right thing or accomplishing their duty while committing crimes, they would indeed demonstrate the delirious nature of drug addiction, and thus the judgment deficiencies it brings about. Levy, relying on Yaffe (2013), nonetheless seems to adopt a more personal definition of “duty” and confuses it with “value.” Drug addicts would not think that they are accomplishing an objectively (normatively) good action, but a good action according to their own values.^34^ Yaffe claims that there is a legal difference between a behavior guided by the agent’s own values and, conversely, one that goes against them. Asking such drug addicts to respect the law is to make them bear too heavy of a burden. Accordingly, they should be findings of diminished responsibility should be available to them.

Again, we doubt the validity of such arguments due to prevailing normative standards. Criminal law currently judges even more harshly people who respect their own values at the expense of respecting the law. Let us recall honor based crimes as examples, or the very definition of misconduct (“*faute*” in French) for that matter. More precisely, let us take the example of provocation in Canada: an accused will be able to argue that the insulting attitude of a soon-to-be ex wife’s new lover amounts to provocation, but will not be able to do the same about a homosexual flirtation, even where the accused is homophobic.^35^ It is indeed hard to obey laws we don’t value. We are nonetheless responsible for disregarding our values to the benefit of those laws.

Despite the weakness of some of Levy’s arguments, it is worth noting the interesting idea they bring about, namely the possibility of clarifying some compulsive disorders and “neurologising” psychiatry (which means to seek to describe psychiatric disorders in terms of organic deficiencies, or on the contrary, establishing psychiatric diagnosis only once the organic causes are excluded). We don’t exclude the possibility that neuroscience could one day demonstrate that drug addiction, or even pedophilia, translates into judgment disorders. They will then have to establish this while keeping in mind the rationalist criteria of criminal law (relation to common reality, ability to distinguish right from wrong, etc.) and addressing typical criticism concerning compulsive behavior, e.g., blame that rises from the fact that no measures were taken by the accused to avoid wrongdoing, even though he or she knew about his or her condition (a kleptomaniac could warn the shop owner, the drug addict could ask for help, a pedophile could avoid working in kindergartens, etc.).

### Cognitive Biases

The “reasonable person” standard is often used in criminal law when objectively assessing the accused’s *mens rea*. It generally serves in cases of omissions rather than actions^36^, since for the former it is harder to evaluate the presence of a clear intention. Indeed, some acts speak for themselves, and we can almost intuitively guess the intention behind them. However, for others, when the accused actually did “nothing” and let the events occur, it is hard to positively find an intention. To know whether or not an attitude is wrongful, we then imagine “a reasonable person” facing similar circumstances. For example, leaving a toddler to play alongside a staircase could be considered criminal negligence, since any reasonable person could foresee that this is obviously not a good idea that will, in all odds, result in a tragedy.

Some studies reveal daily cognitive biases and suggest that the “reasonable person” standard be amended by such findings. Those studies outline, for example, a natural inclination to be overly confident in one’s own judgments (overconfidence effect, Pallier et al., 2002), to filter information confirming these judgments (confirmation bias, Nickerson, 1998) and to ignore or discard conflicting information (bias against disconfirmatory evidence, Buchy et al., 2007); or even the natural tendency to believe that our successes are our own but that our failures are due to others or to external circumstances (self-serving bias, Shepperd et al., 2008), etc. Otherwise put, the reasonable person might not be that reasonable according to classical standards of rationality (e.g., Gigerenzer and Goldstein, 1996).

This is why Dahan-Katz (2013) criticized the judicial decision in Keech v. Commonwealth (1989). In this case, a driver was driving on the wrong side of a highway, while still believing he was on the right side. He persevered for 8 miles without understanding or paying attention to the other drivers’ warnings, and finally caused a deadly accident. The tribunal found him guilty of manslaughter (different from murder) based on the fact that he should have known that he was driving dangerously. Dahan-Katz nonetheless argues that it is plausible that Keech was influenced by a bias according to which “*where a person is under the impression that a hypothesis is correct, indications to the contrary are not necessarily “rationally” considered—beliefs tend to persevere more than they ought to*”. He should therefore have been relieved of all charges.^37^

This suggestion, although stimulating, seems to ignore that the “reasonable person” standard does not call for perfection. It does not refer to the perfect citizen but to the average person. The accused is not required to have rationally taken into consideration every aspect of the situation, but is rather asked to have considered it as an average person would have. However, severe our biases, and regardless of their effect on our rationality, we all share the same and it is according to this norm that we judge each other. We may indeed have a tendency to overestimate our abilities, but Keech’s strange case nonetheless points to an all but ordinary behavior.

Cognitive neuroscience’s claims in this regard could be more nuanced: it wouldn’t inform the law about human frailty (which the law already takes into account) but would weigh in favour of a change of paradigm, from classical rationality standards (even if mediocre, degraded, or bounded; see Gigerenzer and Selten, 2002), to adaptive rationality criteria (Haselton et al., 2009). Persisting in believing one is right when one is wrong, for example, is considered irrational from a classical standpoint, and yet, is completely legitimate on an evolutionary level in terms of fitness (or cost rationale indicating that it costs more to change for an uncertain benefit than to persist in error) (e.g., Haselton and Nettle, 2006).

It can first be re-affirmed that the classical requirement for banality already acknowledges human biases and weaknesses (all the more so since cognitive psychology deals with biases that we experience on a daily basis). First and foremost, adaptive rationality cannot account for the principle of perfectibility present in and necessary to criminal justice. Classic rationality is referred to in the law as reasonableness in order to be accessible to the average citizen. Under this appellation, it retains the mark of an ideal to strive for, and still asks of people that they do their best to achieve that ideal. Adaptive, or bounded rationality is indifferent to the principle of perfectibility. Concretely, it indicates biases’ functions, but it cannot demand to correct them, since those biases can be viewed as “adaptations.” It would only require from people what they minimally already are (and it could certainly not prognosticate on biases adapted to the future). Only the classical ideal of rationality, inherent to its ideal nature, can call for more. Some may consider it as out-dated or excessively onerous. Yet again, law requires only an average rationality, a degree of reasonableness that is relative to a historical, cultural and punctual context. In doing so, it does not abandon the idea that it is *right* or *good* for humans to strive to respect the law by virtue of their capacities and choices. Cognitive neuroscience, and related disciplines (e.g., cognitive psychology, neuroeconomics) would thus not change (or should not change, depending on our ideological attachment to the principle of perfectibility) the paradigm of the “reasonable person” standard, but would *inform* this paradigm with the objective of providing a scientific basis for understanding what standard of reasonableness a particular person might be held to.

Nonetheless, cognitive biases indicate other avenues than the revision of the reasonable person standard, such as training for judges and juries. These could be useful to warn the latter about potential biases in their judgment and that of others. A famous, but controversial, example is a study supposedly showing that judges render harsher decisions when they’re hungry (Danziger et al., 2011; for critics, see Weinshall-Margel and Shapard, 2011; Lakens, 2017). Another classical example comes from studies on eyewitness testimony. Memory of an event that has been witnessed is highly flexible. Exposing a witness to new information during the interval between witnessing the event and recalling it, can substantially modify what the witness recalls (Loftus and Palmer, 1974). Evaluation of eyewitness evidence should probably be more attentive to this issue. Testimony, although essential and relevant evidence, could be considered less reliable, or at least elevate the burden of proof. Again, cognitive psychology findings provide useful tools, but do not radically transform legal practice. Lawyers, before the rise of psychological evidence about the frailty of our judgment and perceptions, have always proceeded in questioning witness and testimonies’ credibility.

### Sentences and Damages

It has been suggested that judicial sentencing be adapted to methods for monitoring and measuring brain activity, mostly in civil law for calculating moral damages, and in criminal law to individualize sentences.

In civil law, introducing new neuroscientific methods to “quantifying” alleged pain and damages would save time in procedural matters, solving and preventing legal disputes. Moreover, civil law applies a less rigorous burden of proof than criminal law: “the evidentiary rules will not apply in their full rigor, possibly making the admission of such evidence more likely.” Procedural legal practice could thus be transformed more quickly in civil law than in criminal law.

In criminal law, the idea is to go from a retributivist conception of the law where criminals deserve their sentences, to a consequentialist conception of the law where considerations for consequences for the group, deterrence, prevention and treatment prevail. In this framework, supported by many scientists (e.g., Greene and Cohen, 2004; Sapolsky, 2004), the criminal is no longer a guilty person deserving sanctions but a sick individual to cure, and sometimes a simple danger for society to neutralize. Some pretend that sentences would then be more “human.” However, Pardo and Patterson (2013), as well as Morse and Roskies, show that contrary to what we may believe, abandoning merits to justify sentences does not lead to softer sentences. On the contrary, “*(…) most of the most draconian aspects of punishment have been motivated by consequential concerns. Striking examples are recidivist sentencing enhancements, the approval of strict liability crimes, the “war on drugs”… and mandatory minimum sentences. None of these can be retributively justified, and all punish disproportionately to desert*” (Morse and Roskies, 2013). The notion of deserved individual blame acts as a safeguard against the society’s hegemonic temptation for security, in the name of which society is often prone, following a consequentialist approach, to sacrifice individual rights. Neuroscience, although certainly not sufficient to choose between legal conceptions, could nevertheless help us improve sentences in terms of efficiency by refining mental disorders or differential diagnosis. Again, neuroscience would not revolutionize law but improve already well-embedded practices (on the matter of potential future neurolaw revolutions, see Kolber, 2014).

Moreover, they give rise to the age-old ethical questions relating to the moral admissibility of certain physical treatments. Some scientists argue for attenuating immoral behaviors, such as racism and physical aggression, through TMS interventions or psychotropic drugs (Douglas, 2008). On the more consensual end of the scale, Coppola’s propositions (Coppola, 2018) concern the use of predictive neuroscientific tools to evaluate recidivism rates^38^, or the individualization of sentences to fit criminals neurobiology and facilitate social reinsertion.^39^ However, the question that arises here, as it indeed has over the course of the history of criminal law, is to choose whether or not criminals should be corrected by means of physical interventions, or by education, punishment, etc. It has always been possible to cut a thief’s arm, or to chemically castrate sexual delinquents. Sociology also presented itself as a good means to evaluate recidivism (Wootton, 1963). Neuroscience only counts here as another possibility on the long list of potential treatments for criminals [for a parallel drawn between the 1960s aversion therapies, as portrayed in *A Clockwork Orange* (Burge, Kubrick), and new techniques such as DBS and WBS, see McMillan, 2018]. Their admissibility leads the way to the procession of eternal ethical questions: the place for the accused’s consent, physical integrity and identity, autonomy, retributivism and consequentialism, etc. (for a thorough discussion presenting both sides, for and against neurological interventions of criminals, see Birks and Douglas, 2018).

### Enhanced Moral Agents

One original suggestion, instead of supporting a paradigm revolution or neuro-treatment, points toward “moral enhancement.” The literature on this topic has arisen with the advent of new ways of enhancing one’s cognitive capacities (may it be smart drugs, DBS etc.), and mostly deals with the main issue of the ethical permissibility of neurointerventions (see Persson and Savulescu, 2008, 2011, 2013; Harris, 2011; Douglas, 2013). Some authors delve specifically into the nexus between enhanced capacities and legal responsibility, questioning for example the duty to take enhancers in certain contexts (and the corollary liability for omissions), the breach of the standard of care that omitting to take enhancers could amount to, and the legal causal nexus between this type of omission and harm (see Goold and Maslen, 2015, for a discussion on those three points and a refutation that enhancers would give rise to these legal situations). Given the extensive literature on moral enhancement, we will only focus here on the influence of cognitive enhancement in determining criminal responsibility, and accordingly, on the validity of the underlying premise behind most claims relating to enhanced responsibility. That premise goes roughly as follows: if (criminal) responsibility is capacitarian and neuro-interventions can enhance our capacities, then those interventions could lead to an enhanced responsibility. In other words, responsibility would account for hypercapacity.

In a synthetic and systematic manner, Nicole Vincent explores the speculative question of responsibility enhancement, arguing specifically on the validity of the aforementioned premise (Vincent, 2013). She first exposes daily cases of responsibility assignments that follow greater capacities: when, for example, we say to a particularly mature child that disappoints us: “I expected more of you.” She then answers 8 objections against the argument that responsibility accounts for hypercapacity, and demonstrates that enhanced capacities could lead to greater responsibility. Enhanced individuals could then be “*expected to satisfy higher standards… and they may even be deemed negligent or reckless for failure or refusal to do so, and possibly even sanctioned*” (Vincent, 2013, p. 329).

Intriguing though that idea may be, it is not immune from criticism. First, criminal responsibility, although capacitarian, is not *proportional* to an individual’s capacities. Responsibility is attributed once certain criteria have been met: it is a threshold, not a scale^40^. The difference in severity across sentences is explained by the absence of certain criteria and not a partial fulfillment thereof. An act, in law, can be characterized as both “voluntary” and not intentional, but not as half voluntary and half intentional (such as manslaughter in Canada, corresponds to voluntary acts of violence without the intention of killing). The same goes for attenuating or aggravating circumstances: those only come into play once *mens rea* has been established. Against this objection, Vincent contends that although responsibility is a threshold, that threshold could be elevated through new cognitive enhancement techniques. Indeed, the “reasonable person” standard has evolved over time. There is a “reasonable person” for every place and time. To know what a future reasonable person will be for the western world is a sociological rather than legal question. In the hypothesis that the future reasonable person would have multiple brain implants, criminal law would remain unchallenged. Only the social norm would have changed. It is also worth noting that this new norm would only concern cases of objective responsibility (i.e., cases of omissions) and that actions would still be assessed through the lens of subjective responsibility (i.e., the subjective abilities to have a feeling of agency, to distinguish right from wrong, etc.). Finally, such an enhancement of the responsibility threshold, does not confirm, as Vincent seems to suggest, that “*responsibility tracks hypercapacity*,” but only that “*responsibility tracks capacity*” (which is a totally uncontroversial statement). Therefore, the so-called enhancement would not be considered enhanced at all, being the new standard.

Secondly, if we allow ourselves to speculate on a responsibility that would be proportional to capacities, we could only observe the disastrous and unfair consequences of such a notion. To be able to judge over-capable, or under-capable, individuals responsible for negligence, we need an objective standard to compare them to. There could not be the “reasonable person” single standard anymore, but a myriad of standards, the “more” or “less,” “little” or “very” reasonable person. The multiplication of standards contradicts de facto the principle of equality in law, and would lead to segregated judicial orders for different classes of population. Besides, the matter of diagnosing hypercapacity remains delicate. Such a diagnosis could not be done at trial, since the accused would thereby never know the applicable standard until their first encounter with the law. Should we then test people every year during the whole of their lives in the eventuality that they be criminally charged? How would such tests work? Although this is somehow theoretically possible – for example, through behavioral modeling of developmental trajectories (e.g., Palminteri et al., 2016) –, it problematically ties such standards to the ungraspable, if not arbitrary, rhythm of scientific progress. Considering that the judges and the jury would have to always be on point concerning science’s evolution, this policy seems impracticable.

To conclude, and extend beyond the initial scope of this section (i.e., focusing on the statement according to which criminal responsibility tracks hypercapacity), let us note that a version of enhanced responsibility already exists in our societies. Ministers, bosses, military superior officers are all people carrying a heavier responsibility tied to their functions. The weight of responsibility in those cases does not flow from greater capacities, but rather from the authority they exercise. Some of the literature on “moral enhancement” suggests that stronger individuals on a neurological level would be vested with some special authority and responsibility in their interactions with others. Neurological strength, however, gives you an authority that is primarily intimate, and not social: you exercise it on yourself, not on others. Should we then “biologise” the notion of authority in such a way that it extends to capacity? Would we not thereby void its meaning as a social influence that we accept at the expense of a greater vulnerability to society’s demands? Is it not fairer for a social institution, by which people judge each other, to lie in the choices individuals make in relation to one another? To her credit, Vincent recognizes the importance of choices (that she addresses through the angle of *consent* to responsibility). She nonetheless considers them as one out of many aspects of responsibility. In this regard, we disagree: criminal responsibility, at least in liberal democracies, is rooted in a social contract. Individual choices are not simple considerations, but the very foundations (and/or justification) of it all.

The question that remains, and indeed is a constant concern, for theorists of enhanced responsibility is this: should foundations of responsibility be “neurologised”? It seems obvious that we regard judging each other’s actions as something that is beneficial to society, but what of judging each other’s biological make up?

## Conclusion

In the course of our analysis, we have defined criminal responsibility as an essentially practical concept independent from free will and other metaphysical questions. Hence, criminal responsibility is immune from debates on determinisms and their affiliated answers. We have recalled that the current and retributivist model of criminal responsibility affords a central place to the individual in relation with the sentence. While asking for an individual’s reasons to act, it treats that individual as a person who deserves blame, but also dignity. Questioning a person’s reasons to act and feeling of responsibility also serves the purpose of evaluating the fairness of the conditions given by society for making a choice. That model is anchored in current popular beliefs regarding accountability and the promotion of certain values. If traditional neuroscience disciplines want to revolutionize law, they cannot simply establish facts. On their own, without any ideological aim, they cannot substantially modify normative practices. They must also engage in a political justification of the changes being called for, convince populations, and take into consideration the ensuing consequences. In turn, this approach must acknowledge and deal with technical, interpretative and legal obstacles that limit the uniform application of neuroscience. Far from a revolution, neuroscience proves to be more beneficial when entering in a subtle dialogue with the law in order to assist the truth-seeking function of the courts. In other words, neuroscience’s greatest potential with respect to the law lies less in assessing the degree of responsibility of an accused than in reconstructing a state of affairs and determining what the implications of that state of affairs may be with respect to the accuracy of allegations.

While neurolaw often evokes the neuroscientification of law, it could more properly refer to the juridification of neuroscience, i.e., legal thinking that would integrate and apply scientific discoveries to criminal justice.

## Author Contributions

AB and VC wrote the manuscript with equal contributions.

## Conflict of Interest Statement

The authors declare that the research was conducted in the absence of any commercial or financial relationships that could be construed as a potential conflict of interest.

## Footnotes

1 H.L.A. Hart, a renowned philosopher of law, emphasized this ambiguity in a famous excerpt on a captain of a ship responsible (or not responsible) in so many ways of so many different things (see Hart, 1968, p. 211).

2 Hereafter we will often take the model of Canadian Criminal law – which we are most familiar with –, but its structure is also present in a number of other legal systems. The need for both a material and a psychological element of the crime to define responsibility, as well as the main exonerations, are indeed found in most, if not all, of the western-inspired legal systems. To have a glance at national criminal law systems, see: International Encylopedia for Criminal Law, ed. by Dr. Frank Verbruggen and Dr. Vanessa Franssen, online : http://www.kluwerlawonline.com/toc.php?pubcode=CRIM. We also note that international criminal law is built upon the same basic categories of material and psychological elements of the crime and exonerations such as mental disorder, intoxication, necessity, duress, self-defense, etc. (see: art. 30 and 31 of the Rome Statute).

3 Such a categorisation is borrowed from Parent (2008).

4 Note that the definition of free will is contentious in itself. According to Frankfurt, an agent is “free” if he wants what he wants, such that his lower-order desires correspond to higher-order volitions (e.g., Frankfurt, 1988). For others (Descartes, Berkeley, Kant), free will requires that an agent can genuinely escape the causal necessity of a deterministic world.

5 The debate on criminal responsibility is independent from the discussion on (moral) naturalism, that is from knowing whether the law, as a system of established rules, derives from our moral *intuitions* or not. We also recall that we only deal with criminal responsibility, without delving into drawing the lines of intersection between law and morality, e.g., assessing the question of whether or not moral responsibility is also independent from free will or if it is intertwined with criminal responsibility.

6 As the next section (and next paragraph) will show, assessment of this subjective experience is also normative.

7 In this regard, one notes that criminal law contextualizes the accused’s actions “in the ordinary course of events” – what “ordinary” means being left to a political (social, cultural, etc.) appreciation.

8 In this case, the interpretation comes from Wegner himself. The logic is the following: since it has been observed that a pre-motor potential (or readiness potential) occurs about 600 ms before conscious awareness of intention, which in turn occurs about 200 ms before action onset, the belief that we intentionally cause our actions (in other words that “consciously willing the action” causes the initiation of action) would be an illusion.

9 This echoes a recent argument from Krakauer et al. (2017) in favor of *behaviourally* driven neuroscience: neuroscience needs behavior to make sense of neural findings. As a matter of fact, the *neural* implementation of behavior is always better investigated after having first carefully studied (i.e., theoretically and experimentally decomposed) the behavior itself (Krakauer et al., 2017; see also infra, “Technical limitations”).

10 *543 U.S. 551*, 2005.

11 S.J. Morse, with humor, considers such arguments as “*the signs of a disorder that I have preliminarily entitled Brain Overclaim Syndrome*” (Morse, 2006, p. 397).

12 The citizen needs to know the law so as to be able to comply with it, and this is made easier when the law is stable and does not follow the rather tumultuous course of scientific advances (see Hu et al., 2018). For example, and as illustrated by the recent Replicability Crisis, a variety of legally relevant notions in cognitive science (e.g., social priming, third-party punishment, biases in judicial decisions) might need to be profoundly revised, if not abandoned (e.g., respectively, Lakens, 2017; Schimmack et al., 2017; Pedersen et al., 2018).

13 Byrne was a violent psychopath who mutilated, raped and killed a young woman in a youth hostel. The Court of Appeal defined the *abnormality of mind* as including the lack of ability to exercise will power to control physical acts in accordance with rational judgment. The Court held that the accused was in such an abnormal state of mind that he did not have the required *mens rea* for murder (the charge was reduced to manslaughter). However, the evidence of abnormality, according to Wootton, relied mainly “*the revolting circumstances of the killing and the subsequent mutilations* ” as well as on “*his previous sexual history*” (Wootton, 1963). See R. v. Byrne (1960) *2 QB 396*.

14 Morse (2019), in fact, seems less optimistic than we are: “*if a criminal defendant behaves rationally in a wide variety of circumstances, the defendant is rational even if his or her brain appears structurally or functionally abnormal. In contrast, if the defendant is clearly psychotic, then a potentially legally relevant rationality problem exists even if his brain looks normal. We might think that neuroscience would be especially helpful in distinguishing the truth in “gray area” cases in which the behavioral evidence is unclear. For example, is the defendant simply very grandiose or actually delusional? But unfortunately, the neuroscience helps us least when we need it the most, and if the behavior is clear, we don’t need it at all*”.

15 A number of articles have interpreted this result as signifying that dogs *understand* human words because lexical processing is associated with a similar pattern of activation in the left hemisphere in most humans (but see also Andics et al., 2017, *Erratum for the Report* “*Neural mechanisms for lexical processing in dogs*”).

16 Among other examples, there are inconsistencies in brain areas associated with moral reasoning: utilitarian decisions (sacrificing one life to save three others) in the Trolley dilemma recruits a structure located in medial part of the prefrontal cortex (the anterior cingulate cortex), while it has been shown that damage to prefrontal regions increases the frequency of utilitarian decisions (Capestany and Harris, 2014).

17 The fact that in linear classification (the method used by most decoding techniques) there is little constraint on how information is selected and classified is both the strength and weakness of the technique. This explains why classifiers can robustly decode features in brain regions that are yet known to code poorly for these features (e.g., visual motion in V1, Seymour et al., 2009; Wang et al., 2014) or can decode arbitrary univariate fMRI signals that classical activation-based analyses could not detect (e.g., Davis and Poldrack, 2013).

18 See also Nahm et al. (2017): “*Large amounts of brain mass and its organic structures, even entire hemispheres, can be drastically altered, damaged, or even absent without causing a substantial impairment of the mental capacities of the affected persons*”. About a patient with hemispherectomy, “*not only does [the patient] perform motor and sensory functions for both sides of the body, [he] performs the associative and intellectual functions normally allocated to two hemispheres*” (Nahm et al., 2017).

19 A non-consensual participant needs only move his or her head slightly to render the results uninterpretable. Thus, it has been consistently shown that subject motion in fMRI produces spurious but systematic correlations in functional connectivity, which are interpreted as true correlations while they are in fact simple motion artifacts (e.g., Power et al., 2012; Van Dijk et al., 2012). Note that the same remark applies with twitches, blinks and fidgets, as important generators of ongoing neural activity (Drew et al., 2018).

20 “*Given that the types of actions at issue in the free will and moral responsibility literature are often preceded by deliberation and are actions according to which we evaluate the agent, the lack of these features in the experiment might seem unsatisfactory.*”

21 For example, driving carefully, and yet above the speed limit, when a snowstorm prevents the driver from seeing the road signs.

22 The act is intentional, but constrained. This type of excuse acknowledges the presence of *mens rea*: in *the Nicomachean Ethics*, Aristotle illustrates the situation of a mixed act by using the image of a captain’s ship in a storm who must abandon his shipment to save his crew. In this case, the captain’s action results from the captain’s choice, and hence it is still a voluntary action even though the action was constrained by external causes.

23 For example, the notion of self-defense is sometimes used to illustrate a claim about responsibility, including in cautious and relevant articles (e.g., Haggard, 2017).

24 Rule 104 Fed. R. Ev. : “*104 (a) Preliminary Questions of Admissibility, and (b) Relevancy Conditioned on Fact, as follows: (a) The court must decide any preliminary question about whether a witness is qualified, a privilege exists, or evidence is admissible. In so deciding, the court is not bound by evidence rules, except those on privilege; (b) When the relevance of evidence depends on whether a fact exists, proof must be introduced sufficient to support a finding that the fact does exist. The court may admit the proposed evidence on the condition that the proof be introduced later.”*

25 Rule 702, Fed. R. Ev.; [*Daubert v. Merrell Dow Pharmaceuticals, Inc., 43 F 1311 (9th Cir. 1995)*, s. d.]

26 All information from this paragraph comes from an excellent procedural review on the question provided by Kaufmann (2013).

27 For a detailed comparative law study, see Spranger (2012).

28 According to Meijer et al. (2013), in the seminal line of research from Farewell and collaborators, only two studies were peer-reviewed – that is to say, 3 datasets with a total of 30 participants (i.e., Farwell and Donchin, 1991; Farwell and Smith, 2001).

29 The P300 component reflects the subject’s *beliefs* rather than the recognition of real facts – but even false memories can return positive results (Satel and Lilienfeld, 2013).

30 This point echoes the critique raised about the use of fMRI scans in judicial settings, and the risk of evidential circularity (see supra, “Interpretative limitations“). As pointed out by a influential neuroscience blogger: “*What do we do about someone whose brain “lights up” to the taboo stimuli (child, or pro-terror), but who denies feeling any attraction? What about someone who acknowledges a taboo desire, but who has never acted upon it and who says they never will? Neuroscience might offer a source of information, but we’d still have to make sense of that data*,” i.e., to refer to the actual behavior (Neuroskeptic, “Do We Need A Neuroscience of Terrorism?”, Discover magazine). A similar remark is made by Coppola (2018): “*There can be cases in which individuals who experience paedophilic urges [and display neurobiological profile associated with paedophilic traits] are still able to resist them.*”

31 This right applies in the United States, Canada, Wales, England, and India.

32 Art. 232(2) C.cr. (Canada).

33 Note that the defense of intoxication can be raised for crimes of specific intent (e.g., murder).

34 Indeed, various studies have shown that pathological gambling is associated with a specific pattern of subjective preferences, characterized by a shift toward risky options (e.g., Ligneul et al., 2013). It should be noted that the interpretation of the word “duty” made by Yaffe derives from a distortion of the common word “value”. A preference for risk-seeking strategies is not axiological.

35 Respectively: *R c. Thibert*, [1996] 1 R.C.S. 37, 52, and *R. c. Tomlinson*, [1998] S.J. (Quicklaw) n848 (Q.B.).

36 The standard of a reasonable person does not only apply to omissions, it also applies to many active offenses, such as the reasonable foreseeability requirement in aggravated assault, or the standard of care of the prudent driver in dangerous driving. We overly simplify its application to give the reader a grasp of what this standard is aiming at.

37 Here Katz overstates the explanatory scope of cognitive biases in general: a confirmation bias can explain why an individual perseverates in performing an erroneous behavior, but it does not explain why this behavior has been adopted in the first place, e.g., it cannot account for Keech’s initial decision to drive on the wrong side of the road (at the most it could explain why it lasted this long, but see below for a counter-argument).

38 For example, the level of activity in the ACC might provide specific information as to whether an offender will be rearrested within 4 years of his release (Aharoni et al., 2013). Along the same line, a correlation has been found between reduced amygdala volume and increased risk for committing future violence in both young and adult males (e.g., Pardini et al., 2014; see Glenn and Raine, 2014; Coppola, 2018, for a review). Other important studies (Pustilnik, 2015, for a review) have characterized potential objective neural measures of how much subjective pain a subject is experiencing – which is important because the law’s system of compensation in personal injury cases awards damages for pain based on mostly subjective assessment (Morse, 2019). These studies surely give a hint about the potential contributions that neuroscience may make to law in the future. Note that we do not insist on these potential contributions because the main focus of our paper is the foundation of criminal responsibility rather than the reliability of neuroscience-based methodology for e.g., predicting criminal behavior or better calculating damages.

39 Neuroprediction might thus “*foster the implementation of alternative individualized sentences tending to offenders’ actual social rehabilitation and social reintegration. Notably, neuroprediction could assist criminal justice systems to integrate current punitive policies and measures with socio-rehabilitative strategies, which could ultimately improve crime prevention and public safety without undermining the individual rights of offenders (…). An example of how neuroscience proves helpful in rehabilitative sentencing comes from Canada, where neurofeedback treatment programs have been tested on juvenile offenders*” (Coppola, 2018).

40 This threshold is relatively simple to reach and does not require extraordinary morality (an ability to distinguish between right and wrong, to perceive the world correctly, to act according to one’s own intentions, etc.).
